# Comparison of simulations with PHITS and HIBRAC with experimental data in the context of particle therapy monitoring

**DOI:** 10.1093/jrr/rrt151

**Published:** 2014-03

**Authors:** Heide Rohling, Lembit Sihver, Marlen Priegnitz, Wolfgang Enghardt, Fine Fiedler

**Affiliations:** 1 Technische Universität Dresden, OncoRay, Dresden, Germany; 2 Applied Physics, Chalmers University of Technology, Gothenburg, Sweden; 3 Helmholtz-Zentrum Dresden-Rossendorf, Dresden Germany

**Keywords:** simulation, particle therapy PET, PHITS, HIBRAC

## Abstract

Therapeutic irradiation with protons and ions is advantageous over radiotherapy with photons due to its favorable dose deposition. Additionally, ion beams provide a higher relative biological effectiveness than photons. For this reason, an improved treatment of deep-seated tumors is achieved and normal tissue is spared. However, small deviations from the treatment plan can have a large impact on the dose distribution. Therefore, a monitoring is required to assure the quality of the treatment. Particle therapy positron emission tomography (PT-PET) is the only clinically proven method which provides a non-invasive monitoring of dose delivery. It makes use of the β^+^-activity produced by nuclear fragmentation during irradiation. In order to evaluate these PT-PET measurements, simulations of the β^+^-activity are necessary. Therefore, it is essential to know the yields of the β^+^-emitting nuclides at every position of the beam path as exact as possible. We evaluated the three-dimensional Monte-Carlo simulation tool PHITS (version 2.30) [ 1] and the 1D deterministic simulation tool HIBRAC [ 2] with respect to the production of β^+^-emitting nuclides. The yields of the most important β^+^-emitting nuclides for carbon, lithium, helium and proton beams have been calculated. The results were then compared with experimental data obtained at GSI Helmholtzzentrum für Schwerionenforschung Darmstadt, Germany. GEANT4 simulations provide an additional benchmark [ 3]. For PHITS, the impact of different nuclear reaction models, total cross-section models and evaporation models on the β^+^-emitter production has been studied. In general, PHITS underestimates the yields of positron-emitters and cannot compete with GEANT4 so far. The β^+^-emitters calculated with an extended HIBRAC code were in good agreement with the experimental data for carbon and proton beams and comparable to the GEANT4 results, see [ 4] and Fig. 1. Considering the simulation results and its speed compared with three-dimensional Monte-Carlo tools, HIBRAC is a good candidate for the implementation in clinical routine PT-PET.

**Fig 1.**
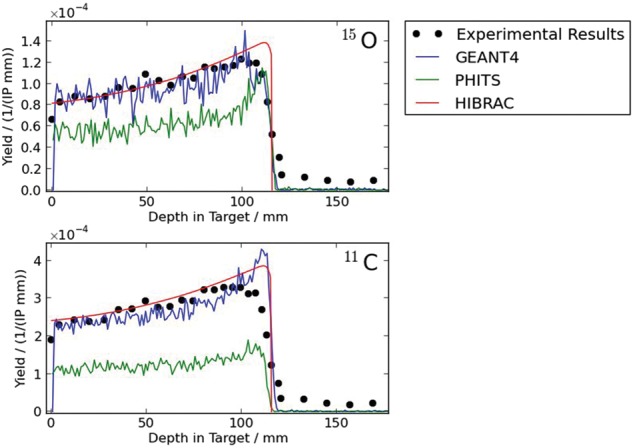
Depth-dependent yields of the production of ^11^C and ^15^O during proton irradiation of a PMMA target with 140 MeV [ 4].

Depth-dependent yields of the production of ^11^C and ^15^O during proton irradiation of a PMMA target with 140 MeV [ 4].
